# Supplementary material for: Structural rearrangements allow nucleic acid discrimination by type I-D Cascade
Source: Nat Commun. 2022 May 20;13:2829. doi: 10.1038/s41467-022-30402-8 (PMC9123187; doi:10.1038/s41467-022-30402-8)

Supplementary Information

Structural basis for PAM-dependent recognition of nucleic acids by type I-D Cascade

Evan A. Schwartz, Tess M. McBride, Jack P.K. Bravo, Daniel Wrapp, Peter C. Fineran, Robert D. Fagerlund, and David W. Taylor

Supplementary Table 1: Plasmids used in this study.

Plasmid	Description	Reference
pSEVA251	Replicative plasmid, RSF1010 replicon, KmR	(Silva-Rocha et al., 2013)
pSEVA1810	pBAD/AraC promoter, pUC replicon, ApR	(Silva-Rocha et al., 2013)
pACYCDuet-1	Two T7/LacO promoters with P15A replicon, CmR	Novagen
pPF1549	N-His$_6$-tagged Cas10d, Cas7d, and Cas5d, pQE-80LoriT	(McBride et al., 2020)
pPF1552	N-His$_6$-tagged Cas6d, Spacer1 of CRISPR array, pACYCDuet-1	(McBride et al., 2020)
pPF1590	pcloDF13, pBAD30	This study
pPF1609	Spacer 1 of the type I-D array flanked by 5′-GTT-3′ PAM, pBAD/AraC promoter, pBAD30	This study
pPF1610	Spacer 1 of the type I-D array flanked by 5′-AAC-3′ PAM, pBAD/AraC promoter, pBAD30	This study
pPF1719	pBAD/AraC promoter, pSEVA251	This study
pPF2451	N-His$_{10}$ tag, pACYCDuet-1	This study
pPF2452	N-His$_{10}$ tagged Cas5d, Cas6d, Cas11d, pACYCDuet-1	This study
pPF2453	N-His$_{10}$ tagged Cas5d, Cas6d, Cas11d, spacer 1 of CRISPR array, pACYCDuet-1	This study
pPF2455	Cas10d, Cas7d, pSEVA251	This study
pPF3021	Modified pPF2455 with Cas10d(K326P)	This study
pPF3023	Modified pPF2455 with Cas10d(K326A)	This study
pPF3026	Modified pPF2455 with Cas10d(ΔS432-Y437)	This study
pPF3025	Modified pPF2453 with Cas5d(K114A)	This study

Supplementary Table 2: Oligonucleotides used in this study.

Name	Sequence (5'-3')	Notes (PAM and PFS bolded)	Restriction site (underlined)
PF2937	AGTCCATATGTTTCCCCGTAAGGGGTCGGAGG	F repeats and spacer 1	NdeI
PF2938	ATGCGGTACCAAGATGGCACTAGATACTAACTCAAACC	R repeats and spacer 1	KpnI
PF3079	/5IRD800CWN/UCACUAGAUCUCGUGCCCACACCCCCGCUUUCCCUUUCAGCUCAGCACAAUUCGACGUAU	NS ssRNA, IRDye800	-
PF3089	CTAGTATCAC**GTT**GATTGTTGTGCCCCTGGCGGTCGCTTTCAATGCCTCGATCC	F 5′-GTT-3′ PAM protospacer	SpeI
PF3090	TCGAGGATCGAGGCATTGAAAGCGACCGCCAGGGGCACAACAATC**AAC**GTGATA	R 5′-GTT-3′ PAM protospacer	XhoI*
PF3091	CTAGTATCAC**AAC**GATTGTTGTGCCCCTGGCGGTCGCTTTCAATGCCTCGATCC	F 5′-AAC-3′ PAM protospacer	SpeI
PF3092	TCGAGGATCGAGGCATTGAAAGCGACCGCCAGGGGCACAACAATC**GTT**GTGATA	R 5′-AAC-3′ PAM protospacer	XhoI*
PF3158	/5IRD700/CGCAACTCTCTACTGTTTCTCCATACC	F dsDNA probes, IRDye700	-

PF3160	/5IRD700/CTCCTCGCCCTTGCTCACCATAAG	R dsDNA probes, IRDye700	-
PF3167	/5IRD800CWN/GCAUGACGGAUCGAGGCAUUGAAAGCGAC CGCCAGGGGCACAACAAUC**AAC**GUGAUACUA	5′-AAC-3′ PFS ssRNA,	-
PF3653	TATACCATGGGCCATCACCATCACCATCATCACCATCACCA CGATTACGATATCCCAACG	F His$_{10}$-TEV-linker	NcoI
PF3654	TGTGGATCCGCCTCCACCGCCCTGAAAATACAGGTTTTCG GTCGTTGGGATATCGTAATC	R His$_{10}$-TEV-linker	BamHI
PF4095	/5IRD700/CTCTCTACTGTTTCTCCCCTAG	F dsDNA probes, IRDye700	-
PF4096	/5IRD700/TCGCCCTTGCTCACCATATG	R dsDNA probes, IRDye700	-
PF4099	GTCGACCTCACTAGTATCAC**GTT**GATTGTTGTGCCCCTGG CGGTCGCTTTCAATGCCTCGATCCTCGAGGCATGCGAT	5′-GTT-3′ PAM Duplex dsDNA	-
PF4100	GAUCGAGGCAUUGAAAGCGACCGCCAGGGGCACAACAAU C**AAC**GUGAUACUAG	5′-AAC-3′ PFS ssRNA	-
PF4980	CAGGGCGGTGGAGGCGGATCCATGACAAAAATTTATCGCT GTAAATTAACTCTC	F *cas5d*	BamHI
PF4981	CATAGTATATCTCCTTATTCTGCAGTCATCCATGATTGTTAA CTGAAACCTG	R *cas6d*	PstI
PF4982	GACTGCAGAATAAGGAGATATACTATGACCGAAAAATTGAA ACTGACTAAACG	F *cas11d*	PstI

PF4983	CATTATGCGGCCGCAAGCTTTTAGTTTTGGTTTTGTTGTGCTTCTAGAG	R *cas11d*	HindIII
PF4991	CGAATTCGAGCTCGGTACCAATAAGGAGATATACTATGACAACACTTCTTCAAACTTTGC	F *cas10d*	KpnI
PF4992	CGACGCGGCCGCAAGCTTGCATGCCTAAGACTTTGCCTTCTTCTTACCAC	R *cas7d*	SphI
PF5590	CTCTCTACTGTTTCTCCCCTAGGCCGCGGCCGCGCGAATTCGAGCTCGGTACC**CGT**GATTGTTGTGCCCCTGGCGGTCGCTTTCAATGCCTGCATGCAAGCTTAGGAGGAAAAACATATGGTGAGCAAGGGCGA	gBlock 5′-CGT-3′ PAM protospacer	-
PF5591	/5IRD800CWN/GCAUGACGGAUCGAGGCAUUGAAAGCGACCGCCAGGGGCACAACAAUC**ACG**GUGAUACUA	5′-ACG-3′ PFS ssRNA, IRDye800	-
PF5633	GATGGGGCAGGCTTAAAGGTTTCTCCCCAAAC	F *cas10d* K326A mutant	-
PF5634	GAGAAACCTTTAAGCCTGCCCCATCCCGTTTAAAACCTAC	R *cas10d* K326A mutant	-
PF6197	CGGGATGGGCCAGGCTTAAAGGTTTCTCCCC	F *cas10d* K326P mutant	-
PF6198	CTTTAAGCCTGGCCCATCCCGTTTAAAACCTAC	R *cas10d* K326P mutant	-
PF6205	CAATTTGGTGGGGCAGGTGCAACCAAAAACTATCCG	F *cas5* K114A mutant	-
PF6206	GTTGCACCTGCCCCACCAAATTGAACAGAACG	R *cas5* K114A mutant	-
PF6207	CCAAGTGCAAGGCTGGTATCGGGTTGCAG	F *cas10d* ΔS432-Y437 mutant	-
PF6208	GATACCAGCCTTGCACTTGGGTTTCCTCCGG	R *cas10d* ΔS432-Y437 mutant	-

*Partial restriction enzyme recognition site

Supplementary Table 3: Bacterial strains used in this study.

Strain	Genotype/Phenotype/Description	Reference
DH5α	*E. coli* F^-, ϕ80d*lacZ*ΔM15, Δ(*lacZYA–argF*)U169, *endA1*, *recA1*, *hsd*R17 ($r_K^- m_K^+$), *deoR*, *thi-1*, *sup*E44, λ^-, *gyr*A96, *rel*A1	Gibco/BRL
LOBSTR	*E. coli* B F^- *ompT, gal, dcm, lon, hsdS*$_B$($r_B^- m_B^-$), λ(DE3 [*lacI lacUV5-T7p07 ind1 sam7 nin5*]) [*malB*$^+$]$_{K-12}$(λ^S) *arnA slyD*	Kerafast
Synechocystis sp. PCC 6803	Glucose tolerant laboratory wild-type strain GT-01	[70,71]

Supplementary Table 4: Cryo-EM data collection, map, and model statistics.

	dsDNA-bound Cascade (EMD-24974) (PDB 7SBA)	ssRNA-bound Cascade (EMD-24976) (PDB 7SBB)
**Data collection and processing**		
Magnification	22500	22500
Voltage (kV)	300	300
Electron exposure (e–/Å^2)	41.2	41.2
Defocus range (μm)	-1 to -2.2	-1 to -2.2
Pixel size (Å)	1.045	1.045
Symmetry imposed	C1	C1
Initial particle images (no.)	4,111,214	4,111,214
Final particle images (no.)	336,400	167,273
Map resolution (Å)<br>FSC threshold	2.9<br>0.143	3.1<br>0.143
Map resolution range (Å)	2.6 to >7	3.1 to >9
**Refinement**		
Model resolution (Å)<br>FSC threshold	3.3<br>0.5	3.5<br>0.5
Model resolution range (Å)	N/A	N/A
Map sharpening *B* factor (Å^2)	N/A	N/A
Model composition<br>Non-hydrogen atoms<br>Protein residues<br>Ligands	 60189<br>3669<br>0	 56232<br>3415<br>0
B factors (Å^2)<br>Protein<br>Nucleotide	 43.90/171.82/73.36<br>43.61/124.23/70.66	 43.21/196.00/77.48<br>44.49/102.27/71.18
R.m.s. deviations<br>Bond lengths (Å)<br>Bond angles (°)	 0.013<br>2.02	 0.013<br>2.112
**Validation**<br>MolProbity score<br>Clashscore<br>Poor rotamers (%)	 1.58<br>1.84<br>2.75	 1.95<br>5.06<br>3
Ramachandran plot<br>Favored (%)<br>Allowed (%)<br>Disallowed (%)	 95.69<br>4.20<br>0.11	 95.35<br>4.50<br>0.15

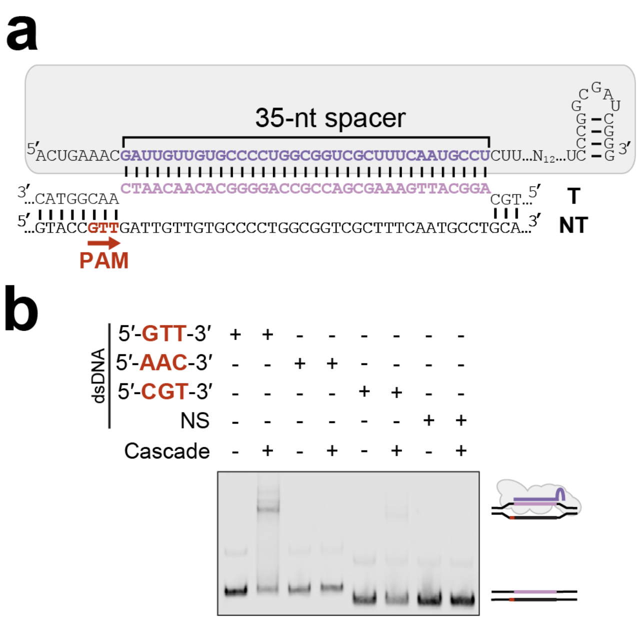

Supplementary Fig. 1: PAM-dependent dsDNA binding. a, Schematic representation of type I-D Cascade (grey protein and purple crRNA) binding to complementary target sequence (protospacer;pink). **b,** Representative mobility shifts of fluorescently labeled protospacer dsDNA with various three nucleotide protospacer adjacent motif (PAM), 5′-GTT-3′ 5′-AAC-3′, and 5′-CGT-3′, and a non-specific (NS) probe on the NT strand, with or without 400 nM type I-D Cascade. Three independent assays were performed. Uncropped gel image is provided as Supplementary Data 1.

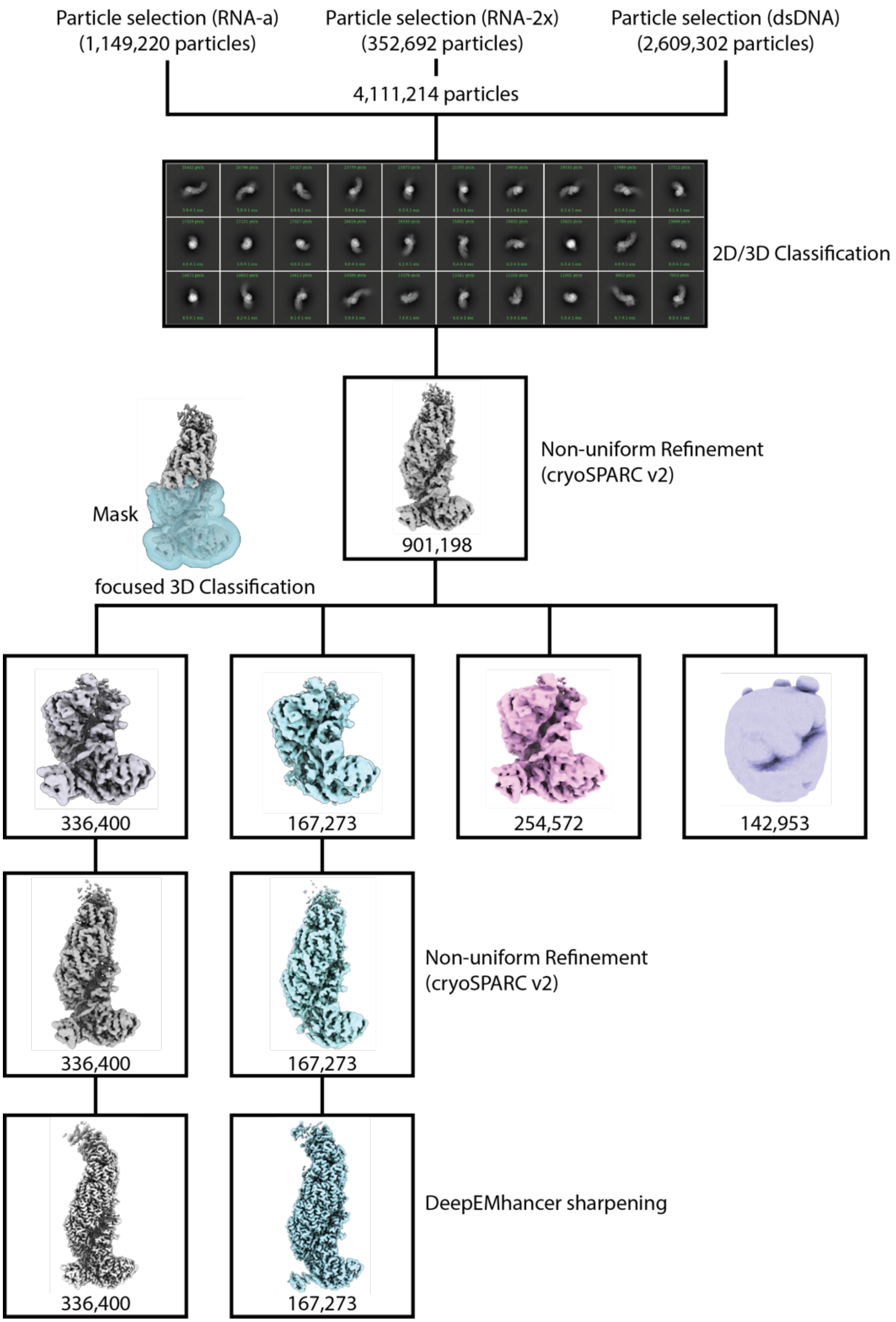

Supplementary Fig. 2: Simplified cryo-EM data processing workflow. The full workflow is described in **Methods**.

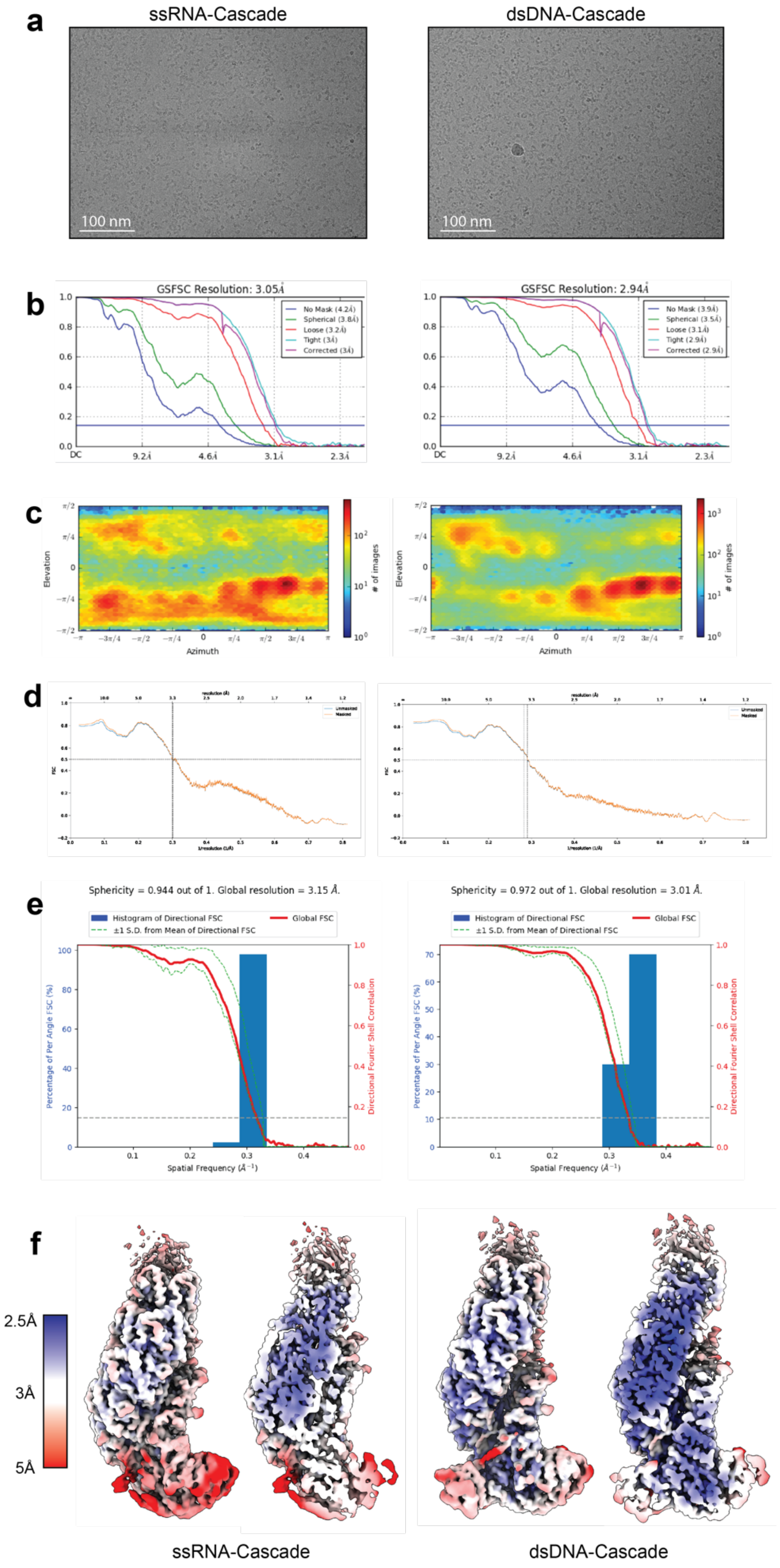
a
ssRNA-Cascade
dsDNA-Cascade
100 nm
100 nm
b
GSFSC Resolution: 3.05Å
GSFSC Resolution: 2.94Å
No Mask (4.2Å)
Spherical (3.8Å)
Loose (3.2Å)
Tight (3Å)
Corrected (3Å)
No Mask (3.9Å)
Spherical (3.5Å)
Loose (3.1Å)
Tight (2.9Å)
Corrected (2.9Å)
c
Elevation
Azimuth
# of images
d
e
Sphericity = 0.944 out of 1. Global resolution = 3.15 Å.
Sphericity = 0.972 out of 1. Global resolution = 3.01 Å.
Histogram of Directional FSC
±1 S.D. from Mean of Directional FSC
Global FSC
Percentage of Per Angle FSC (%)
Directional Fourier Shell Correlation
Spatial Frequency (Å^{-1})
f
2.5Å
3Å
5Å
ssRNA-Cascade
dsDNA-Cascade

Supplementary Fig. 3: Statistics and structure validation of the two cryo-EM maps. a, Motion corrected cryo-electron micrographs of the ssRNA-Cascade and dsDNA-Cascade data collection. **b**, FSC plot of the two maps based on the 0.143 gold standard of two half maps. **c**, Euler angular distribution plot showing orientation of the particles that contributed to the final 3D model. **d**, Map-to-Model resolution for the dsDNA model (left) and ssRNA model (right) at an FSC of 0.5. **e**, Sphericity plot showing the spatial frequency of each model. The range of one standard deviation is represented in a dashed green curve. **f**, The two final maps colored by local resolution.

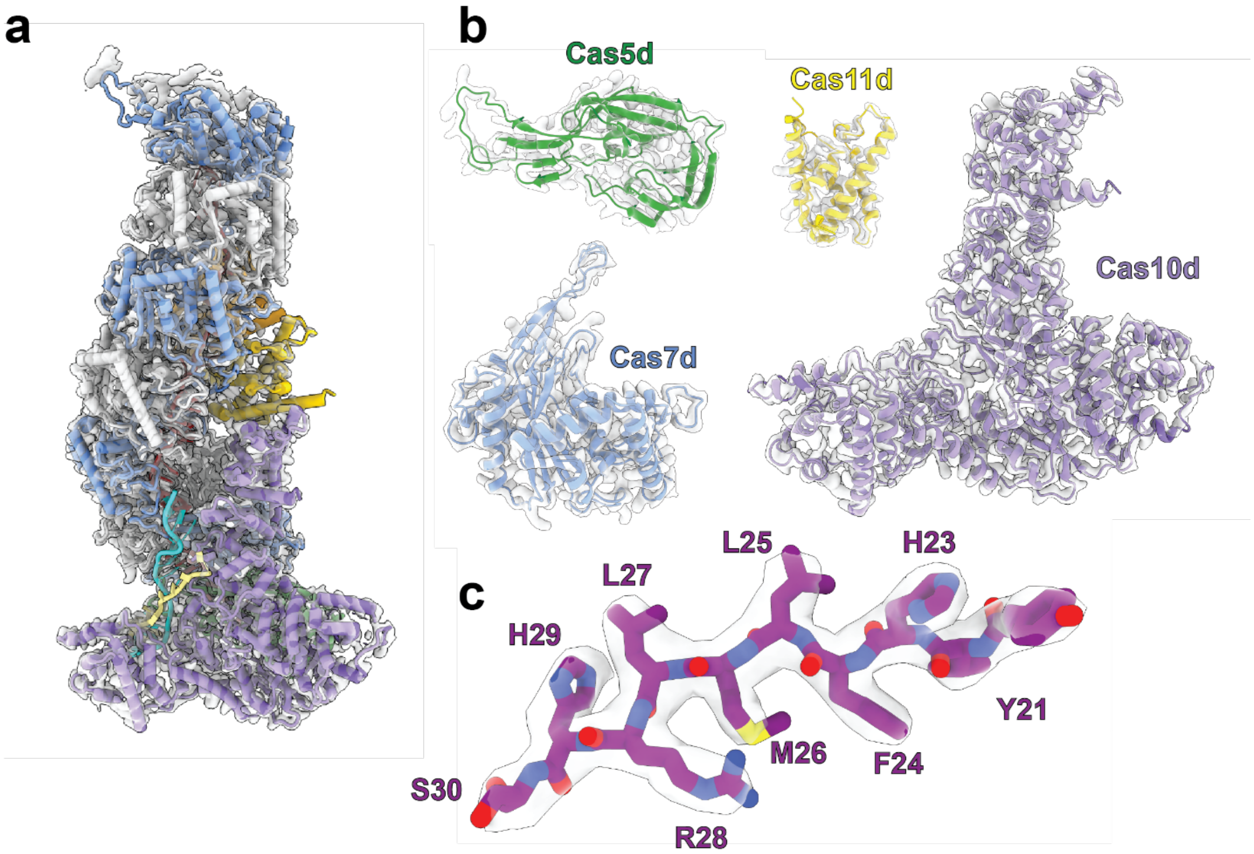

Supplementary Fig. 4: Type I-D Cascade EM map quality. a, Model of the full type I-D Cascade fit into the EM map. **b,** Individual subunits fit into their corresponding density. **c,** Beta strand within Cas7d shown to represent resolution quality within the map.

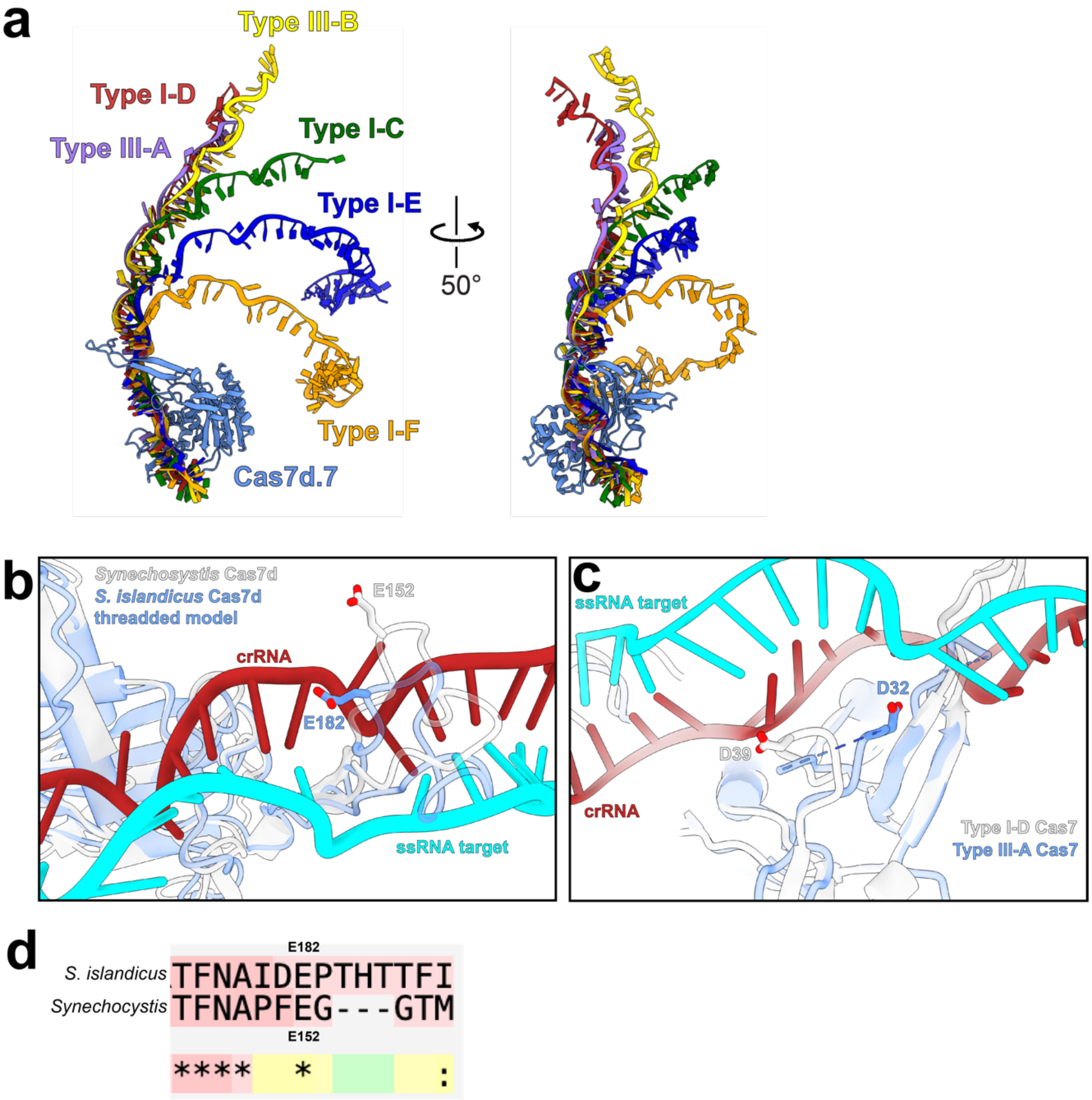

Supplementary Fig. 5: crRNA geometry and Cas7 comparison between various type I and type III systems. a, CRISPR RNA geometries overlaid by aligning the Cas7 subunits of each subtype. The type I-D crRNA angular geometry appears to align better with the type III-A and type III-B crRNAs than any type I crRNA. The range of crRNA angles provides a potential delineation of the evolution between these CRISPR subtypes. **b**, Comparison of the *S. islandicus* Cas7d sequence threaded onto our *Synechocystis* Cas7d model. The predicted active residue of E182 aligns both structurally and in sequence space, as shown in **d**. Neither residue is positioned to cleave the kinked ssRNA target. **c**, Comparison of the active residue D32 from the type III-A system and D39 from type I-D. D39 does not appear to be in a position to cleave the ssRNA target, while D32 does.

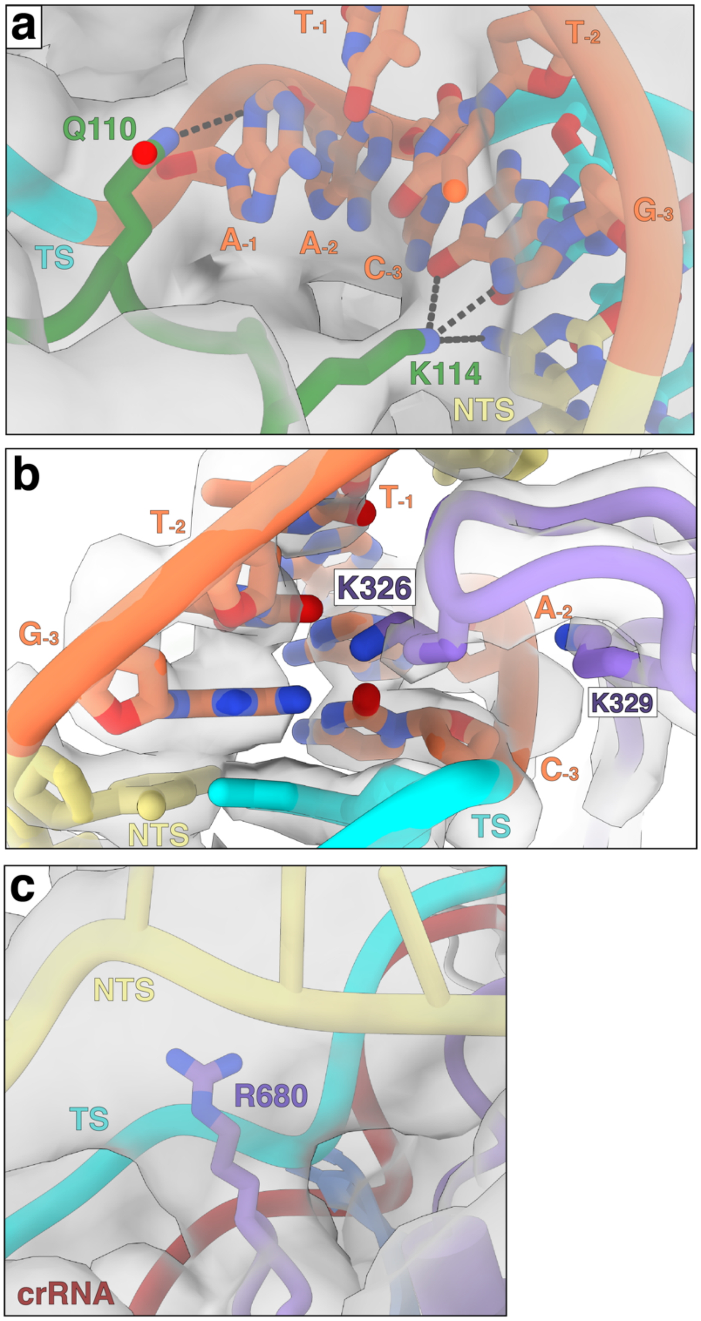

Supplementary Fig. 6: Cas10d and Cas5d contacts with the dsDNA target. a, Q110 and K114 of Cas5d interact with PAM bases A (-1 position) of the TS and G (-3 position) of the NTS, respectively. **b,** Representative EM density for the PAM recognition residue, K326, and the PAM duplex. **c,** R680 of Cas10d is one of the residues that contacts the NTS backbone to guide the NTS towards the Cas10d HD site.

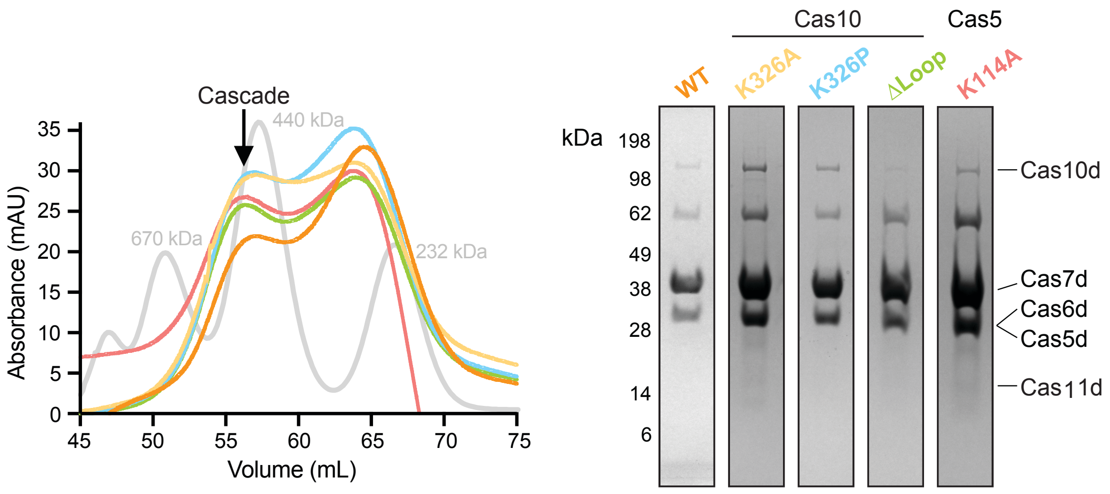

Supplementary Fig. 7: Purification of I-D Cascade with mutations in Cas10d and Cas5d residues that contact with the dsDNA target. **a,** Size exclusion chromatography profile of the I-D Cascade PAM-interacting mutants. Source Data is provide as Source Data file. **b,** SDS-PAGE analysis of each I-D Cascade mutant, collected at the 55 mL elution volume from the size exclusion chromatography profile in **a.** Uncropped gel images are provided in Supplementary Data 2.

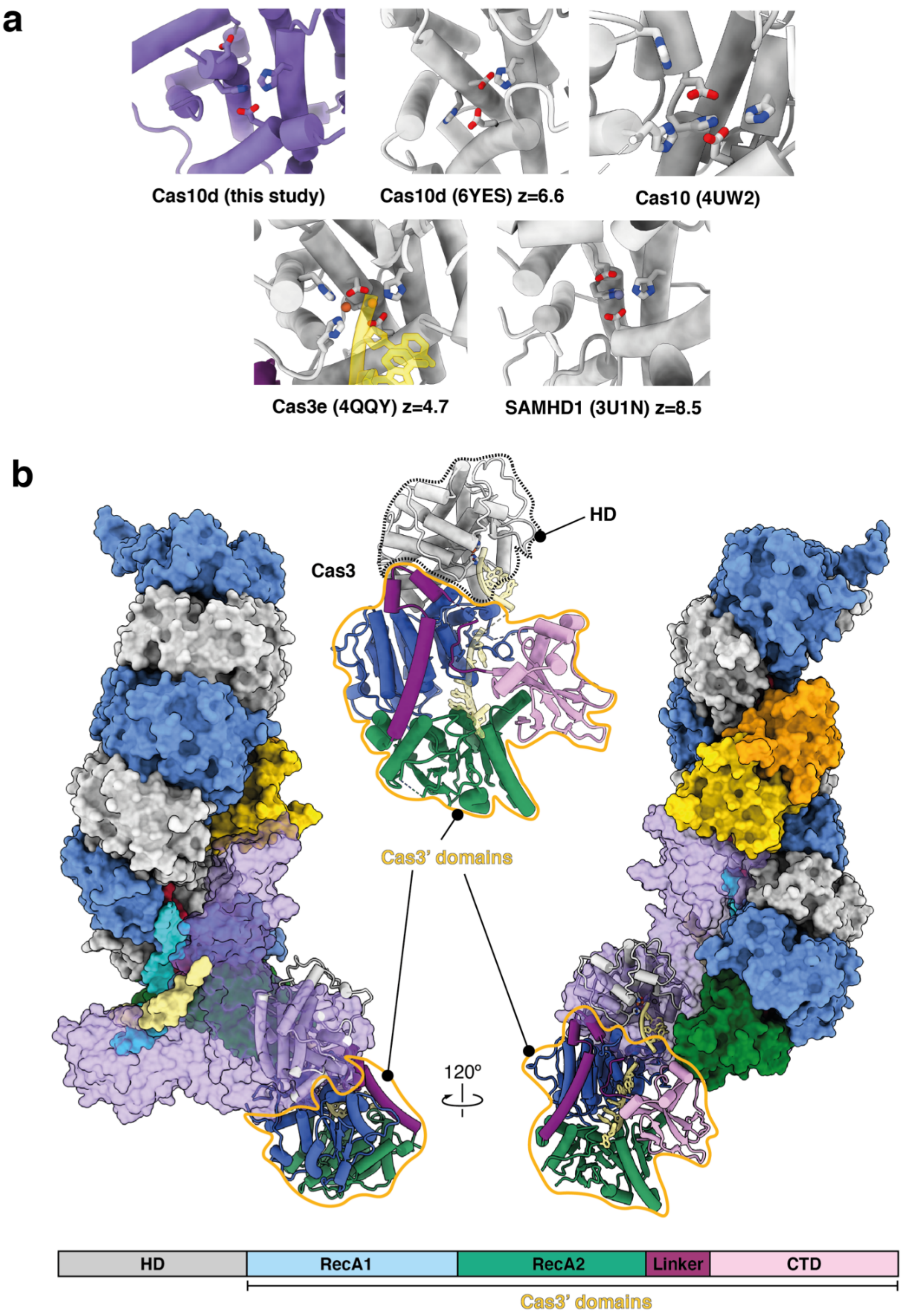

Supplementary Fig. 8: HD Comparison and Cas3′ docking prediction. a, HD comparison between *Synechocystis* Cas10d, *S. islandicus* Cas10d, *T. onnurineus* Type III-A Cas10, *T. fusca* Cas3e, and *H. sapiens* SAMHD1. Z scores for the structural overlap are listed. SAMHD1 was

included since it was the best structural homologue (i.e. had the highest Z-score) to Cas10d HD domain and has a representative HD domain active site. Type III Cas10 structures are typically poorly resolved around the HD domain, and therefore were not identified as structurally similar to our Cas10d model. The type III-A Cas10 has no Z-score due to a lack of significant structural homology to our Cas10d HD domain. **b**, Cas3e HD domain overlayed onto the Cas10d HD domain. The Cas3e helicase domains interestingly protrude out exactly where Cas3′ may bind.

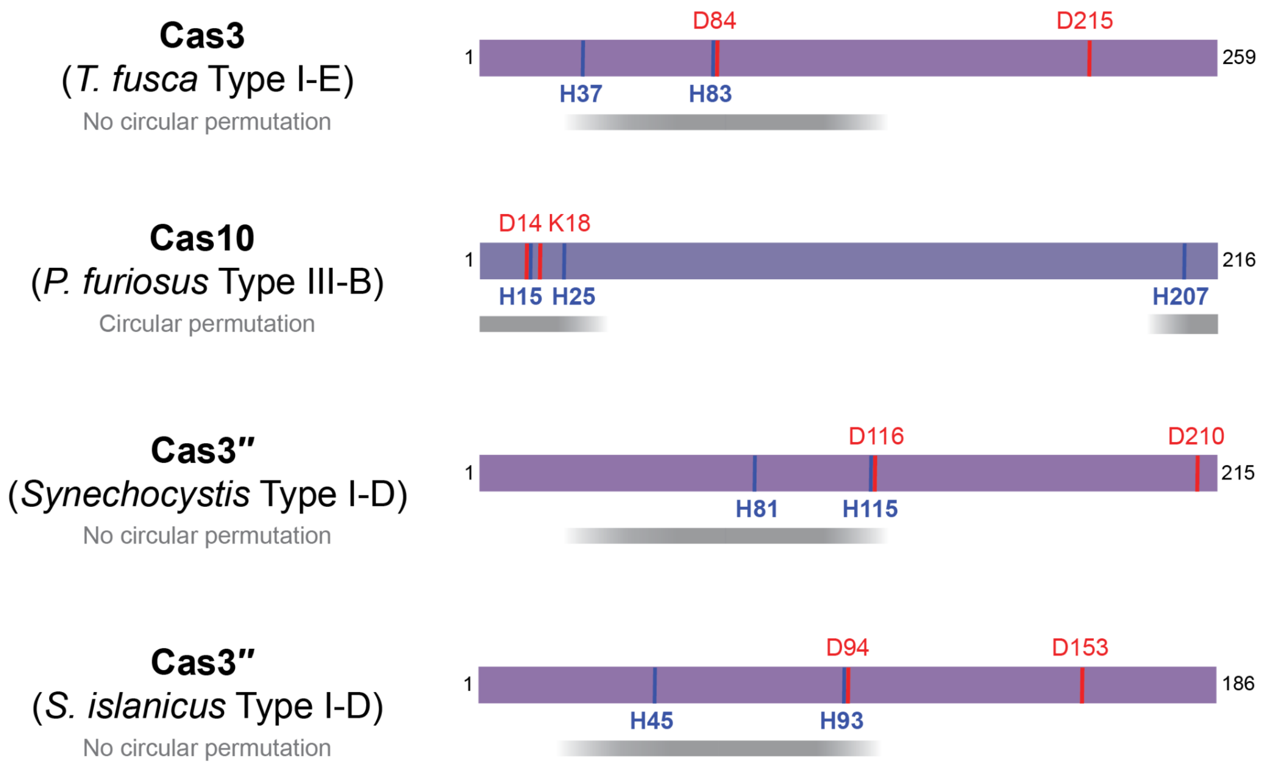

Supplementary Fig. 9: Ion coordinating histidines within the HD domains in type I (Cas3) and type III (Cas10) systems. Histidines within the HD domain comparison between *T. fusca* type I-E Cas3, *P. furiosus* type III-B Cas10, *Synechocystis* type I-D Cas3′′, and *S. islandicus* type I-D Cas3′′. Position of histidines (grey shading) indicative of a circular permutation (shading split in two) or no circular permutation (shading clustered). Numbers indicate amino acid position within the protein.

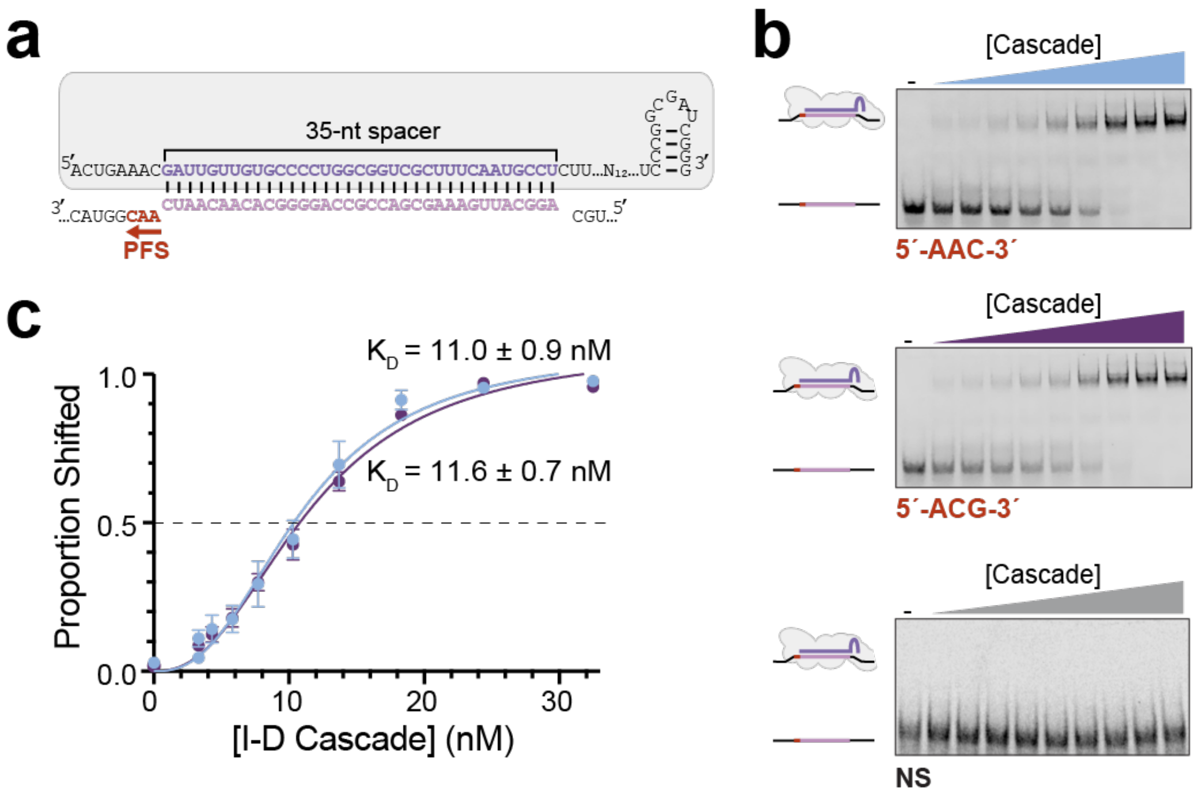

Supplementary Fig. 10. Role of PFS in ssRNA binding a, Schematic representation of type I-D Cascade (grey protein and purple crRNA) binding to complementary target sequence (protospacer;pink). **b,** Representative mobility shifts of fluorescently labeled protospacer ssRNA with various three-nucleotide protospacer flanking sequences (PFS): 5′-AAC-3′ (blue), 5′-ACG-3′ (purple), and a non-specific (NS) probe (grey) with increasing amounts of type I-D Cascade ranging from 3 to 33 nM in 1.33x increments. Three independent assays were performed. **c,** Dose-response curves of the proportion of shifted-probe as a function of type I-D Cascade concentration. The blue curve corresponds to the 5’-AAC-3’ PFS probe and the purple curve corresponds to the 5’-AGC-3’ PFS probe. Data are represented as mean ± SEM. The apparent dissociation constants (K_D) are based on three independent assays. A K_D could not be determined for the non-specific RNA target (panel **b**, bottom). Source Data are provided as a Source Data file.

Supplementary Data

Supplementary Data 1. Uncropped gel image for **Supplementary Fig. 1b**.

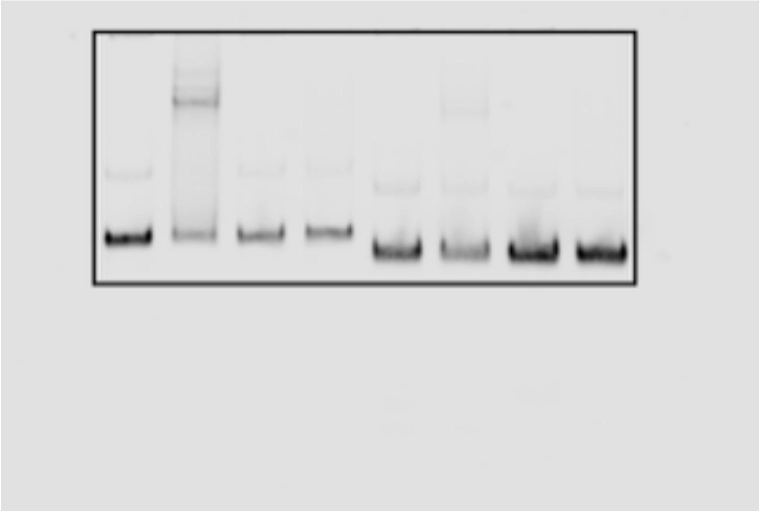

Supplementary Data 2. Uncropped gel images for **Supplementary Fig. 7**.

WT SDS-PAGE

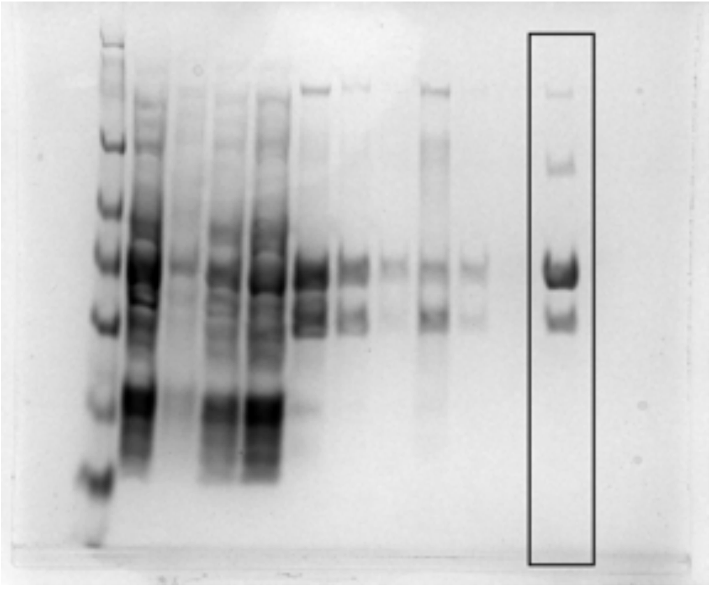

K326A SDS-PAGE

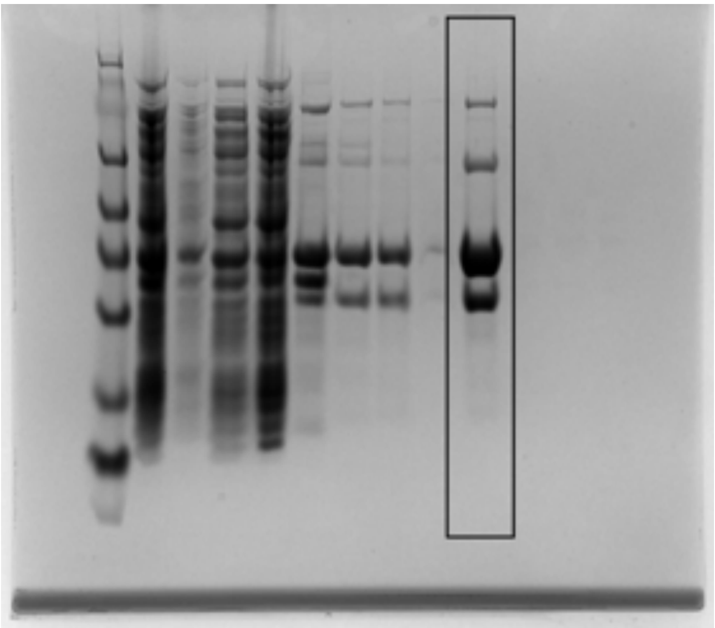

K326P SDS-PAGE

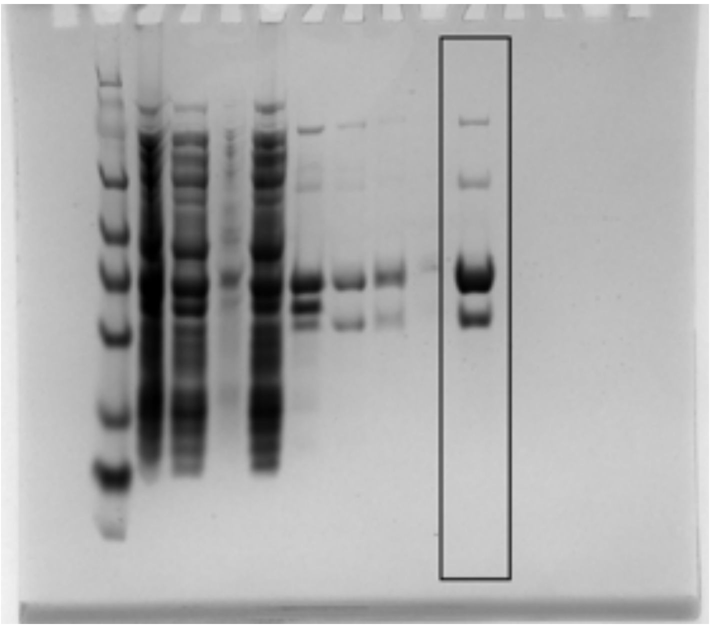

ΔLoop SDS-PAGE

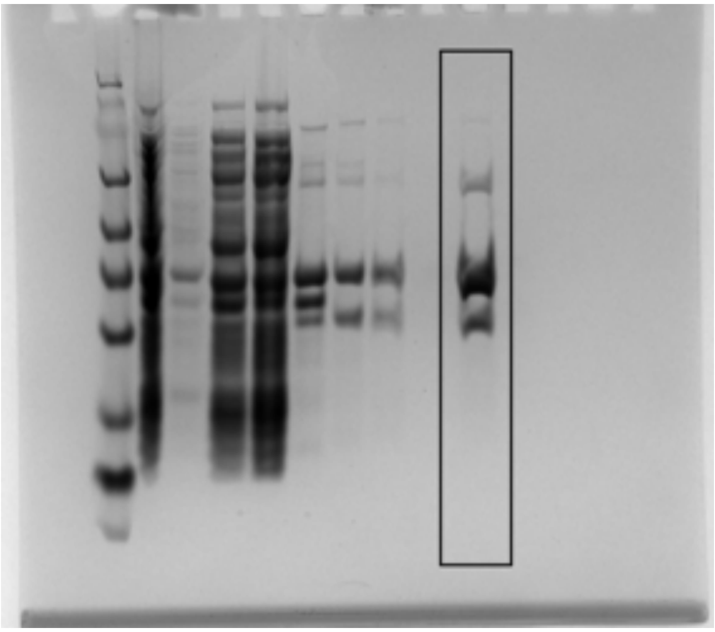

K114A SDS PAGE

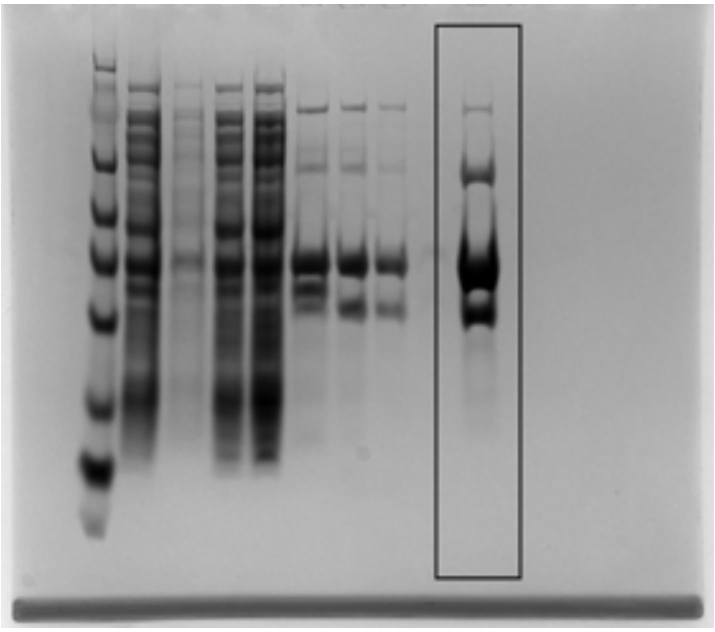

Supplement: Supplementary file 1 — Supplementary Information [file 41467_2022_30402_MOESM1_ESM.pdf]
