# Supplementary material for: Structural rearrangements allow nucleic acid discrimination by type I-D Cascade
Source: Nat Commun. 2022 May 20;13:2829. doi: 10.1038/s41467-022-30402-8 (PMC9123187; doi:10.1038/s41467-022-30402-8)
Supplement: Supplementary file 3 — Reporting Summary [file 41467_2022_30402_MOESM3_ESM.pdf]

nature portfolio

Corresponding author(s): David W. Taylor

Last updated by author(s): Apr 9, 2022

# Reporting Summary

Nature Portfolio wishes to improve the reproducibility of the work that we publish. This form provides structure for consistency and transparency in reporting. For further information on Nature Portfolio policies, see our Editorial Policies and the Editorial Policy Checklist.

## Statistics

For all statistical analyses, confirm that the following items are present in the figure legend, table legend, main text, or Methods section.

| n/a | Confirmed | |
|---|---|---|
| ☐ | ☒ | The exact sample size (*n*) for each experimental group/condition, given as a discrete number and unit of measurement |
| ☐ | ☒ | A statement on whether measurements were taken from distinct samples or whether the same sample was measured repeatedly |
| ☒ | ☐ | The statistical test(s) used AND whether they are one- or two-sided<br>*Only common tests should be described solely by name; describe more complex techniques in the Methods section.* |
| ☒ | ☐ | A description of all covariates tested |
| ☒ | ☐ | A description of any assumptions or corrections, such as tests of normality and adjustment for multiple comparisons |
| ☐ | ☒ | A full description of the statistical parameters including central tendency (e.g. means) or other basic estimates (e.g. regression coefficient) AND variation (e.g. standard deviation) or associated estimates of uncertainty (e.g. confidence intervals) |
| ☐ | ☒ | For null hypothesis testing, the test statistic (e.g. *F*, *t*, *r*) with confidence intervals, effect sizes, degrees of freedom and *P* value noted<br>*Give P values as exact values whenever suitable.* |
| ☐ | ☒ | For Bayesian analysis, information on the choice of priors and Markov chain Monte Carlo settings |
| ☐ | ☒ | For hierarchical and complex designs, identification of the appropriate level for tests and full reporting of outcomes |
| ☐ | ☒ | Estimates of effect sizes (e.g. Cohen's *d*, Pearson's *r*), indicating how they were calculated |

*Our web collection on statistics for biologists contains articles on many of the points above.*

## Software and code

Policy information about availability of computer code

| | |
|---|---|
| Data collection | Data were collected using a Titan Krios cryo-electron microscope operated at 300kV using a Gatan K3 direct electron detect or opera ed in counting mode. |
| Data analysis | Cryo-EM data acquisition was monitored by on-the-fly pre-processing in WARP v1.0.9. Data were further processed using cryoSPARC v3.2 and RELION 3.0. Model building and refinement of the structures were performed using PHENIX v1.18rc5 as well as Namdinator v20191016-5814c947. Models were also manually built in Coot v0.8.9 and Isolde v1.2. Model validation was performed using MolProbity as implemented within PHENIX v1.18rc5. Visualization was performed using ChimeraX v1.0. |

For manuscripts utilizing custom algorithms or software that are central to the research but not yet described in published literature, software must be made available to editors and reviewers. We strongly encourage code deposition in a community repository (e.g. GitHub). See the Nature Portfolio guidelines for submitting code & software for further information.

## Data

Policy information about availability of data

All manuscripts must include a data availability statement. This statement should provide the following information, where applicable:

- Accession codes, unique identifiers, or web links for publicly available datasets
- A description of any restrictions on data availability
- For clinical datasets or third party data, please ensure that the statement adheres to our policy

The cryo-EM structure and associated atomic model of dsDNA-bound Cascade have been deposited into the Electron Microscopy Data Bank and the Protein Data Bank with accession codes EMD-24974 [https://www.ebi.ac.uk/pdbe/entry/emdb/EMD-24974] [ and PDB 7SBA [http://doi.org/10.2210/pdb7SBA/pdb], respectively. The cryo-EM structure of ssRNA-bound Cascade and associated atomic model have been deposited with accession codes EMD-24976 [https://www.ebi.ac.uk/pdbe/

entry/emdb/EMD-24976] and PDB 7SBB [http://doi.org/10.2210/pdb7SBB/pdb], respectively. Source Data are provided with this paper. Materials and correspondence requests should be addressed to David W. Taylor (dtaylor@utexas.edu).

# Field-specific reporting

Please select the one below that is the best fit for your research. If you are not sure, read the appropriate sections before making your selection.

☒ Life sciences ☐ Behavioural & social sciences ☐ Ecological, evolutionary & environmental sciences

For a reference copy of the document with all sections, see nature.com/documents/nr-reporting-summary-flat.pdf

# Life sciences study design

All studies must disclose on these points even when the disclosure is negative.

| | |
|---|---|
| Sample size | Each dataset contained 1.5 to 2.5 million particles, and at least at least 997,043 particles, and at least 167,000 particles were used for the final reconstruction. These are typical image numbers for cryo-EM datasets to obtain high-resolution reconstructions. |
| Data exclusions | 2D and 3D classification procedures were used to exclude damaged and 'bad' particles. This is standard practice in cryo-EM and is necessary in order to obtain homogeneous high-resolution cryo-EM structures. No data were excluded from the biochemical experiments. |
| Replication | Cryo-EM datasets were collected with multiple samples in separate imaging sessions. The majority of the protein components of the complex were nearly identical among structures from all datasets. Three independent biological replicates were performed for the biochemical experiments. |
| Randomization | No randomization was performed. Randomization is not used in cryo-EM structural studies. |
| Blinding | No blinding was performed. Blinding is not relevant to this study. Particle classification and resolution estimation were performed automatically from processing software packages. |

# Reporting for specific materials, systems and methods

We require information from authors about some types of materials, experimental systems and methods used in many studies. Here, indicate whether each material, system or method listed is relevant to your study. If you are not sure if a list item applies to your research, read the appropriate section before selecting a response.

## Materials & experimental systems

| n/a | Involved in the study | |
|---|---|---|
| ☒ | ☐ | Antibodies |
| ☒ | ☐ | Eukaryotic cell lines |
| ☒ | ☐ | Palaeontology and archaeology |
| ☒ | ☐ | Animals and other organisms |
| ☒ | ☐ | Human research participants |
| ☒ | ☐ | Clinical data |
| ☒ | ☐ | Dual use research of concern |

## Methods

| n/a | Involved in the study | |
|---|---|---|
| ☒ | ☐ | ChIP-seq |
| ☒ | ☐ | Flow cytometry |
| ☒ | ☐ | MRI-based neuroimaging |
